# Grief and ruminative thought after perinatal loss among Turkish women: one-year cohort study

**DOI:** 10.1590/1516-3180.2021.0148.R1.09062021

**Published:** 2022-03-14

**Authors:** Ebru Gozuyesil, Ayse Inel Manav, Saliha Bozdogan Yesilot, Mete Sucu

**Affiliations:** I RN, PhD. Associate Professor, Midwifery Department, Faculty of Health Sciences, Cukurova University, Adana, Turkey.; II RN, PhD. Assistant Professor, Nursing Department, Faculty of Health Sciences, Osmaniye Korkut Ata University, Osmaniye, Turkey.; III RN, PhD. Lecturer, Nursing Department, Faculty of Health Sciences, Cukurova University, Adana, Turkey.; IV MD. Associate Professor, Department of Gynecology and Obstetrics, Faculty of Medicine, Cukurova University, Adana, Turkey.

**Keywords:** Abortion, spontaneous, Rumination, cognitive, Stillbirth, Women, Perinatal grief, Rumination, Miscarriage

## Abstract

**BACKGROUND:**

Among women who have suffered loss of pregnancy, the level of grief decreases gradually. Age, mental health status and childlessness are the factors known to mostly affect women’s levels of grief.

**OBJECTIVES:**

To assess the levels of grief among women who experienced perinatal loss and the changes in their ruminative thought styles over the first year after their loss.

**DESIGN AND SETTING:**

One-year follow-up study carried out in a university hospital in Turkey.

**METHODS:**

The study population included 70 women who experienced loss of pregnancy in the hospital. The sample size was calculated using G*Power V3.1. Data were collected at 48 hours, at the third month, at the sixth month and at one year after pregnancy loss, between June 2018 and June 2019. A personal information form, the Perinatal Grief Scale and the Ruminative Thought Style Questionnaire were used for data collection.

**RESULTS:**

The women’s highest levels of grief and ruminative thought style were in the first 48 hours. Their tendency towards grief and ruminative thought styles decreased over the repeated measurements during the follow-up. Women aged 20-29 years had the highest levels of grief at the third month after perinatal loss.

**CONCLUSIONS:**

Nursing assessments regarding grief and ruminative thought style over the first 48 hours after perinatal loss should be integrated into nursing care for these women. Grief follow-up programs for these women can be developed through nursing research.

## INTRODUCTION

Perinatal loss is the loss of a baby prematurely or at a short time after birth. The most common forms of perinatal losses are miscarriage, stillbirth and neonatal death.^1^ Despite all the improvements in medicine, perinatal losses still occur frequently and affect millions of families.^1,2^ It has been estimated that approximately 15%-20% of all pregnancies end in miscarriage or spontaneous abortion within the first 12 weeks, worldwide.^3^ The miscarriage and stillbirth rate in Turkey was 18.6% in 2018.^4^ The perinatal death rate was reported to be 6 in 1000 live births in the United States in 2016; it was 11 in 1000 live births in Turkey in 2018.^5,6^

Perinatal loss is one of the most painful and unbearable life experiences for parents, and it causes emotional responses such as grief and depression. Fear, disappointment, anger, self-pity, feelings of failure, etc., are experienced during the period of grief.^2^ Sex, age, perception of loss, life changes, coping styles, support systems and rumination can affect the duration of grief.^2,7^

Ruminative thought has effects on individuals’ negative responses.^8^ Rumination consists of repetitively thinking through a negative emotion or situation.^9^ Individuals with ruminative thoughts isolate themselves, continually focusing on their problems, and feel the results of negative situations for longer times. They think that, through such behavior, they are trying to find a solution.^10^ Studies have shown that rumination is associated with various psychological problems such as depressive symptoms,^11,12,13,14^ anxiety, worry and symptoms of prolonged grief.^7,13,15^ In the literature, it has been noted that women have ruminative tendencies more than men do.^10,11,13,14,16^

Determining the gradual changes in women’s grief levels and their ruminative thought styles may be the starting point and first step in planning nursing care for individuals who have experienced perinatal loss. In this regard, there is one previous study assessing the level of grief among women with pregnancy loss in Turkey.^17^ To the best of our best knowledge, no studies have evaluated the gradual changes in grief levels and ruminative thought styles among women who suffered perinatal loss in Turkey.

## OBJECTIVE

The aim of this study was to evaluate differences in grief levels and ruminative thought styles among women who have experienced perinatal loss, by means of repeated measurements over the first year after the event.

## METHODS

This was a one-year follow-up study carried out in a university hospital. The study population consisted of women who had experienced pregnancy loss in the hospital. The number of women who used the hospital’s delivery service between April 1 and May 1, 2018, was 49. The sample size was calculated using the G*Power software (version 3.1.9.2; Universität Düsseldorf, Düsseldorf, Germany), in terms of the change in R^2^ in multiple linear regression approximation. The minimum sample size required for seven predictors with 80% power and medium effect size (f^2^ = 0.15) was calculated as 43 subjects.^18,19^ However, through considering abandonment over the course of the repeated measurements of the study, we decided to include a total of 70 women in the study.

The women who were included in this study were voluntary participants who had experienced pregnancy loss in any trimester of pregnancy, and who had the ability to speak and write in Turkish. Women who had previously had a psychological disorder were not included in the study, and women whom the researchers were unable to reach during the repeated follow-ups were excluded.

### Data collection forms and tools

Data were collected using a personal information form, the Perinatal Grief Scale (PGS) and the Ruminative Thought Style Questionnaire (RTSQ).

### Personal information form

This form was prepared by the researchers. It consisted of 10 questions regarding sociodemographic and obstetric characteristics.

### Perinatal grief scale

The PGS was developed by Toedter et al. and assesses the level of grief experienced after perinatal loss.^20^ The original scale consists of 33 items on a five-point Likert-type scale and includes three subscales.^21^ These three subscales are named Active Grief, Difficulty Coping and Despair. These levels represent progression of the pathological condition on the overall scale. An Overall Grief score of 91 or higher means that grief is present. An Active Grief score of 34, a Difficulty Coping score of 30 and a Despair score of 27 are used as cutoff points on the three subscales. The Cronbach’s α values lie between 0.86 and 0.92. A validity and reliability study was conducted in Turkey by Özgür Köneş et al. in 2017, and its Cronbach’s alpha was found to be 0.95.^22^ In the present study, the Cronbach’s alpha values were between 0.793 and 0.857 for the subscales of the PGS and 0.930 for the total score.

### Ruminative thought style questionnaire

The RTSQ was developed by Brinker and Dozois in 2009, and its adaptation to Turkish culture was published by Karatepe et al., in 2013.^14,23^ This scale has 20 items. Assessments are made based on the total score. The lowest score is 20 and the highest score is 140. Its Cronbach’s alpha in the above studies was 0.907.^14,23^ In the present study, Cronbach’s alpha was 0.886.

### Data collection

Data were collected in four steps from June 2018 to June 2019. In the first step, the questionnaires were completed for the first time just before the participants were discharged. These women had stayed in the hospital for 48 hours (T_0_) after their pregnancy loss. The women’s phone numbers and addresses were obtained at this time.

The same questionnaires were filled out through phone calls at the third month (T_1_) and sixth month (T_2_) and at one year (T_3_) after discharge. The questionnaires at T_0_ were filled out through face-to-face interviews conducted by the researchers. They explained to the participants that later on they would call them by phone, to fill out the same questionnaires again. The first interviews lasted approximately 30 minutes. The repeated interviews lasted approximately 15 minutes each.

**Figure 1.**
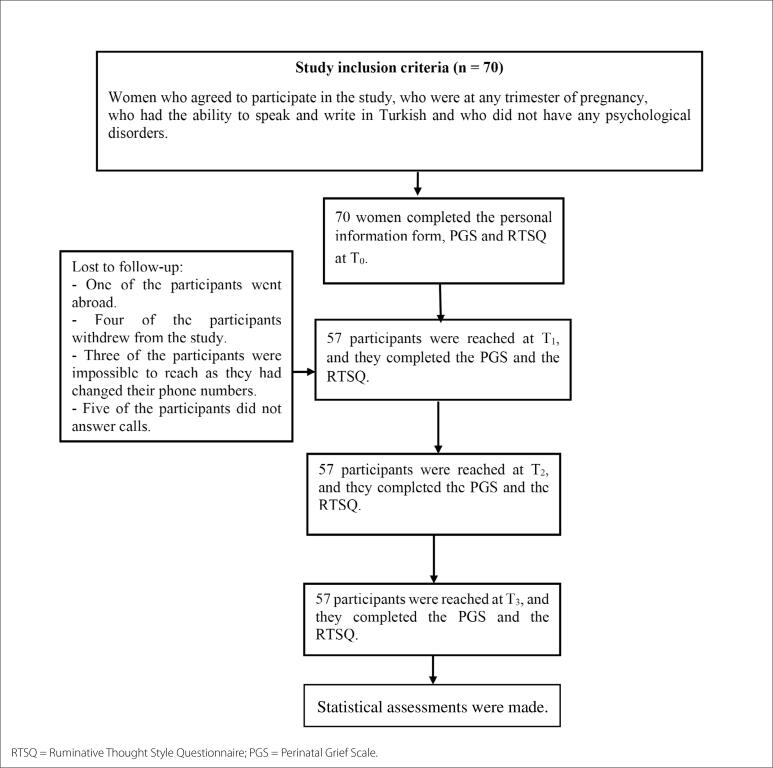
Study flowchart. RTSQ = Ruminative Thought Style Questionnaire; PGS = Perinatal Grief Scale.

At T_0_, 70 participants were involved in the study, but 13 participants did not answer the call at T_1_. The researchers were able to reach all of the T_1_ participants again, at both T_2_ and T_3_, and thus the study was completed with 57 participants. The researchers called all the women who did not answer the phone, at least three times before deciding to drop them from the study. All the details regarding the study process are explained in the study flowchart (Figure 1).

### Data analysis

Statistical analyses were done using the IBM SPSS Statistics software V23 (IBM Corporation, Armonk, New York, United States) and the R Studio software (R Studio, Boston, United States). The normal distribution compliance of the data was examined using the Kolmogorov-Smirnov test. Continuous variables with normal distribution were presented as the mean (standard deviation [SD]); non-normal variables were reported as the median (minimum-maximum [min-max]). Categorical variables were presented as the number of events and percentages. The frequencies of categorical variables were compared using Pearson’s χ^2^ or Fisher’s exact test, as appropriate. The Mann-Whitney U test was used to compare differences between two independent groups when the independent variable was not normally distributed, and Student’s t test was used for normally distributed variables. Repeated measurements of scales were evaluated via the repeated-measurement analysis of variance (ANOVA) test, the Friedman test and nonparametric analysis of longitudinal data in factorial experiments. For experiments with F1-LD-F1 design, Wald-type statistics (WTS) were calculated for testing group and time effects, and interactions. The Kruskal-Wallis test and one-way ANOVA test were used for comparisons of more than two independent groups. Relationships between two continuous variables were tested by means of the Spearman rank correlation. The multiple linear regression-backward elimination technique was used for estimation of RTSQ scores via independent variables. The significance level accepted was P < 0.05.

### Ethical considerations

Ethics approval (number: 2018.02.22/3-10; date: February 22, 2018) was obtained from the University Scientific Research and Publication Ethics Committee of the Osmaniye Korkut Ata University. The procedures used in this study adhered to the tenets of the Declaration of Helsinki. Informed consents were obtained from the participants after we had explained the objectives of the study to them.

## RESULTS

The mean age of the participants was 30.34 ± 6.55 years, and the mean week in which pregnancy loss occurred was 15.42 ± 6.61. Among all the participants, 45.7% had miscarriages and 54.3% had stillbirths. The perinatal deaths all occurred at between 4 and 32 weeks of gestation. Table 1 shows the sociodemographic and obstetric characteristics of the participants.

**Table 1.** Sociodemographic and obstetric characteristics of the participants

	n	%
**Age (years)**		
20-29	31	44.3
30-39	33	47.1
40-49	6	8.6
**Education level**		
Primary school	21	30.0
Secondary school	13	18.6
University and above	36	51.4
**Working status**		
Not working	55	78.6
Working	15	21.4
**Educational level of spouse**		
Primary school	15	21.4
Secondary school	20	28.6
University and above	35	50.0
**Income level perception**		
Poor	5	7.1
Moderate	42	60.0
Good	23	32.9
**Residence**		
Village	9	12.9
District	19	27.1
City	42	60.0
**Family type**		
Nuclear family	56	80.0
Large family	14	20.0
**Number of pregnancies**		
Primiparous	18	25.7
Multiparous	52	74.3
**Childlessness**		
Yes	28	40.0
No	42	60.0
**Number of pregnancy losses**		
One	39	55.7
Two	20	28.6
Three or more	11	15.7
**Number of this pregnancy**		
First	20	28.6
Second	21	30.0
Third	14	20.0
Fourth or more	15	21.4
**Type of loss**		
Miscarriage	32	45.7
Stillbirth	38	54.3

The median values of the total scores on the PGS (P < 0.001) and the mean scores on the RTSQ (P < 0.001) differed over time (Table 2).

**Table 2.** Descriptive statistics on the Perinatal Grief Scale (PGS) and the Ruminative Thought Style Questionnaire (RTSQ)

	Time	χ ± standard deviation	Median (minimum-maximum)	Test statistics	P
**Total PGS**	T_0_^A^	95.94 ± 22.81	93 (43-143)	χ^2^ = 91.363	< 0.001
	T_1_^A^	76.70 ± 20.26	75 (42-130)		
	T_2_^B^	58.40 ± 18.73	57 (36-120)		
	T_3_^B^	53.79 ± 17.98	47 (36-125)		
**Active grief**	T_0_^A^	27.75 ± 7.26	26 (14-44)	χ^2^ = 60.886	< 0.001
	T_1_^B^	25.07 ± 7.62	24 (11-44)		
	T_2_^C^	20.70 ± 7.60	20 (10-40)		
	T_3_^B^	19.12 ± 7.30	16 (10-43)		
**Difficulty coping**	T_0_^A^	36.47 ± 7.96	36 (14-52)	χ^2^ = 92.065	< 0.001
	T_1_^B^	23.18 ± 6.09	23 (13-40)		
	T_2_^C^	18.88 ± 5.05	16 (13-34)		
	T_3_^B^	18.21 ± 5.33	16 (13-41)		
**Despair**	T_0_^A^	34.40 ± 9.20	35 (15-52)	χ^2^ = 118.790	< 0.001
	T_1_^B^	25.77 ± 8.29	25 (11-47)		
	T_2_^C^	18.82 ± 7.65	18 (11-46)		
	T_3_^B^	15.19 ± 6.15	13 (10-38)		
**RTSQ**	T_0_^A^	91.26 ± 25.61	95 (21-140)	F = 8.450	< 0.001
	T_1_^B^	79.51 ± 23.04	82 (20-131)		
	T_2_^C^	79.96 ± 25.44	82 (25-139)		
	T_3_^B^	77.89 ± 24.93	80 (25-132)		

F = repeated analysis of variance; χ^2^= Friedman test statistic; A-C = there are no differences between times that have the same letter for each subscale/total score.

**Figure 2.**
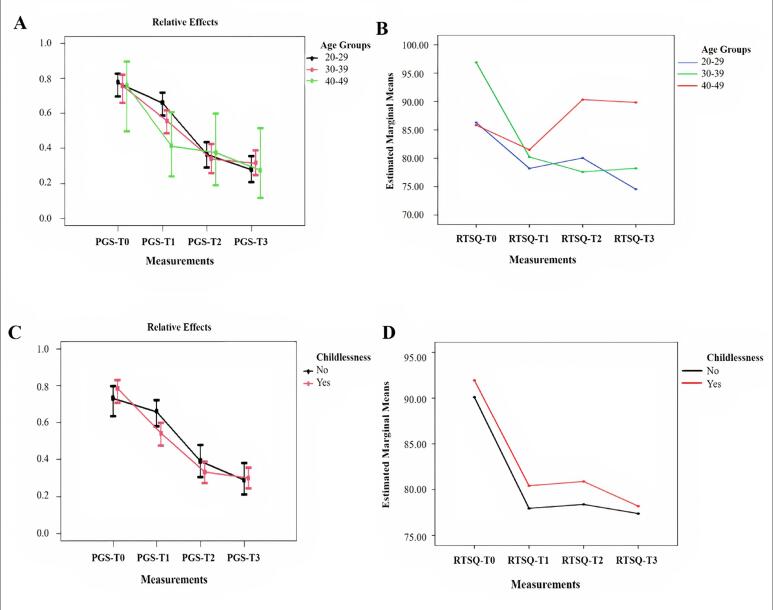
Changes in the age groups and childlessness variables of the Perinatal Grief Scale (PGS) and Ruminative Thought Style Questionnaire (RTSQ) at one-year follow-up: a) age groups of the PGS; b) age groups of the RTQS; c) childlessness of the PGS; D) childlessness of the RTQS.

A statistically significant difference was found between total score median values on the PGS at T_1_ in terms of the variables of age and childlessness (P < 0.05). The difference based on the age variable was caused by the 20 to 29-year age group (P < 0.05). The total median scores on the PGS among women who were unable to have children were higher at T_1_ than at other times (P < 0.05) (Table 3; Figure 2). Figure 2 shows the changes in the variables of age and childlessness on the PGS and RTSQ over the one-year follow-up.

**Table 3.** Distribution of scale scores in terms of some variables over time

	PGS T_0_ Median (min-max)	PGS T_1_ Median (min-max)	PGS T_2_ Median (min-max)	PGS T_3_ Median (min-max)	Statistical significance P-value	RTSQ T_0_ χ ± SD	RTSQ T_1_ χ ± SD	RTSQ T_2_ χ ± SD	RTSQ T_3_ χ ± SD	Statistical significance P-value
**Age (years)**										
20-29	88 (66-138)	77 (50-130)	56.5 (39-105)	48.5 (38-125)	Age: WTS = 1.194 P = 0.551 Time: WTS = 70.631; P < 0.001 Age*Time: WTS = 10.480; P = 0.105	97.11 ± 22.05	81.31 ± 16.42	57.95 ± 16.42	53.09 ± 18.76	Age: F = 0.348; P = 0.708 Time: F = 2.855; P = 0.069 Age*Time: ATS = 1.626; P = 0.180
30-39	96 (43-143)	70 (43-122)	52 (36-120)	44 (36-101)		96.42 ± 23.87	74.51 ± 20.43	58.07 ± 21.13	55.25 ± 18.22	
40-49	101.5 (50-130)	57 (42-83)	52.5 (36-90)	43.5 (39-90)		95.83 ± 28.21	60.83 ± 18.02	59.33 ± 20.70	52.16 ± 19.46	
	χ^2^ = 0.016 P = 0.992	χ^2^ = 7.169 P = 0.028 ^†^	χ^2^ = 0.286 P = 0.867	χ^2^ = 0.157 P = 0.924		F = 0.784 P = 0.484	F = 0.071 P = 0.931	F = 0.607 P = 0.548	F = 0.411 P = 0.904	
**Childlessness**										
Yes	88 (50-143)	80 (50-130)	60 (39-105)	45 (39-125)	Childlessness: WTS = 0.547; P = 0.459 Time: WTS = 135.88; P < 0.001 Childlessness*Time: WTS = 7.730; P = 0.051	88.82 ± 27.45	77.95 ± 26.46	78.38 ± 28.89	77.38 ± 29.62	Childlessness: ATS = 0.112; P = 0.739 Time: ATS = 7.707; P = 0.001 Childlessness*Time: ATS = 0.032; P = 0.955
No	100 (43-138)	71.5 (42-122)	55.5 (36-120)	48 (36-101)		89.80 ± 25.41	80.41 ± 21.13	80.88 ± 23.57	78.19 ± 27.22	
	Z = -1.167 P = 0.243	Z = -2.326 P = 0.020 ^†^	Z = -0.985 P = 0.324	Z = -0.025 P = 0.980		t = -0.154 P = 0.878	t = -0.387 P = 0.701	t = -0.356 P = 0.723	t = -0.118 P = 0.907	
**Number of pregnancy losses**										
One	99 (63-143)	77 (50-130)	60 (37-120)	48 (39-125)	Number of pregnancy losses: WTS = 7.508; P = 0.023 Time: WTS = 42.07; P = < 0.001 Number of pregnancy losses*Time: WTS = 4.445; P = 0.617	90.46 ± 22.05	85.70 ± 22.86	85.09 ± 26.64	81.80 ± 26.34	Number of pregnancy losses: ATS = 2.141; P = 0.127 Time: F = 12.094; P = 0.095 Number of pregnancy losses*Time: ATS = 2.094; P = 0.095
Two	90 (50-138)	71 (42-91)	47 (37-90)	44 (38-90)		80.20 ± 30.68	69.94 ± 20.15	72.17 ± 20.65	72.05 ± 20.65	
Three or more	84 (43-114)	77 (43-97)	52 (36-87)	54 (36-80)		102.45 ± 26.19	76.22 ± 24.20	77.00 ± 27.79	75.44 ± 27.50	
	χ^2^ = 1.116 P = 0.572	χ^2^ = 4.173 P = 0.124	χ^2^ = 3.579 P = 0.167	χ^2^ = 0.928 P = 0.629		F = 2.799 P = 0.068	F = 2.859 P = 0.066	F = 1.516 P = 0.229	F = 0.887 P = 0.418	
**Type of loss**										
Miscarriage	94.5 (50-143)	73.5 (42-117)	57 (36-90)	48 (36-90)	Type of loss: WTS= 3.065; P = 0.079 Time: WTS = 18.04; P < 0.001 Type of loss*Time: WTS = 0.579; P = 0.761	91.50 ± 25.22	79.98 ± 23.21	80.31 ± 25.55	78.21 ± 25.05	Type of loss: ATS = 0.136; P = 0.714 Time: ATS = 3.374; P = 0.044 Type of loss*Time: ATS = 0.016; P = 0.975
Stillbirth	92 (43-130)	75 (47-130)	55 (37-120)	45 (38-125)		89.17 ± 31.28	75.50 ± 23.04	77.00 ± 26.59	75.17 ± 26.00	
	Z = -0.343 P = 0.731	Z = -1.366 P = 0.172	Z = -0.415 P = 0.678	Z = -0.240 P = 0.810		t = -0.332 P = 0.741	t = 0.615 P = 0.541	t = 0.083 P = 0.934	t = -0.444 P = 0.659	

PGS = Perinatal Grief Scale; RTSQ = Ruminative Thought Style Questionnaire; min-max = minimum-maximum; SD = standard deviation; χ^2^ = Kruskal-Wallis test; Z = Mann-Whitney U test; F = one-way analysis of variance (ANOVA) test; ATS = repeated-measurement ANOVA test; WTS = Wald-type statistics, t: Student’s t test; ^†^P < 0.05.

**Figure 3.**
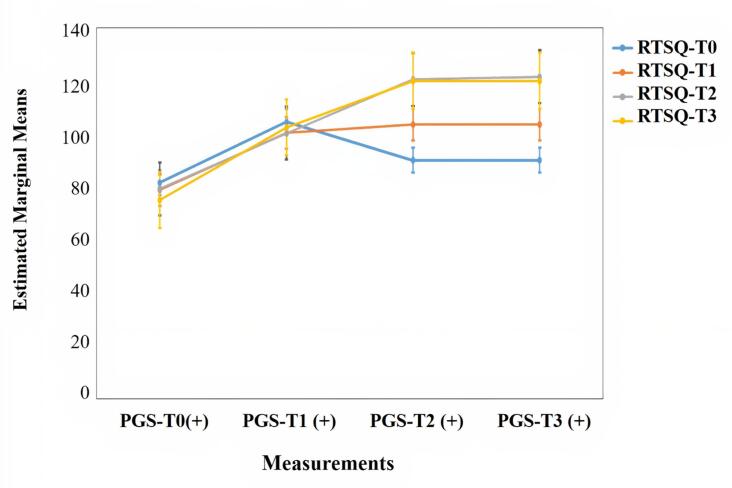
The changes in the Ruminative Thought Style Questionnaire (RTSQ) scores based on the Perinatal Grief Scale (PGS); cutoff points at one-year follow-up.

According to the correlation results from the scales, positive medium-level correlations were found between the total scores on the RTSQ and the PGS and their subscales at T_0_, T_1_ and T_2_ (P < 0.05) (Table 4). Also, it was found that the percentages of the women who had PGS total scores ≥ 91 were 55.7% at T_0_, 21.1% at T_1_, 3.5% at T_2_ and 3.5% at T_3_. A score higher than this cutoff point means having grief. The changes in the RTSQ scores based on the PGS cutoff points over time are shown in Figure 3.

**Table 4.** Correlation analyses on the scales

		Active grief	Difficulty coping	Despair	Total PGS
**T_0_**					
Difficulty coping	r	**0.481**			
	P	**< 0.001**			
Despair	r	**0.697**	**0.787**		
	P	**< 0.001**	**< 0.001**		
Total PGS	r	**0.798**	**0.855**	**0.957**	
	P	**< 0.001**	**< 0.001**	**< 0.001**	
Total RTSQ	r	**0.373**	**0.309**	**0.395**	**0.461**
	P	**0.001**	**0.009**	**0.001**	**0.001**
**T_1_**					
Difficulty coping	r	**0.666**			
	P	**< 0.001**			
Despair	r	**0.811**	**0.734**		
	P	**< 0.001**	**< 0.001**		
Total PGS	r	**0.898**	**0.853**	**0.912**	
	P	**< 0.001**	**< 0.001**	**< 0.001**	
Total RTSQ	r	**0.369**	**0.288**	**0.401**	**0.360**
	P	**0.005**	**0.030**	**0.002**	**0.006**
**T_2_**					
Difficulty coping	r	**0.583**			
	P	**< 0.001**			
Despair	r	**0.888**	**0.667**		
	P	**< 0.001**	**< 0.001**		
Total PGS	r	**0.943**	**0.748**	**0.961**	
	P	**< 0.001**	**< 0.001**	**< 0.001**	
Total RTSQ	r	**0.338**	0.246	**0.323**	**0.303**
	P	**0.010**	0.065	**0.014**	**0.022**
**T_3_**					
Difficulty coping	r	**0.474**			
	P	**0.000**			
Despair	r	**0.836**	**0.510**		
	P	**0.000**	**0.000**		
Total PGS	r	**0.939**	**0.641**	**0.928**	
	P	**0.000**	**0.000**	**0.000**	
Total RTSQ	r	**0.306**	0.124	0.240	**0.289**
	P	**0.021**	0.357	0.072	**0.029**

PGS = Perinatal Grief Scale; RTSQ = Ruminative Thought Style Questionnaire; r = Spearman correlation.

The multiple linear regression-backward elimination technique was used for RTSQ score estimation. The regression model included family type, childlessness, working status and PGS T_3_. PGS T_3_ contained active grief, difficulty coping and despair. Dummy variables were created for categorical variables in the model. Active grief and family type were found to be statistically significant in the regression model (P < 0.05). They explained 15.6% of the change in RTSQ score (Table 5).

**Table 5.** Regression model for estimation of Ruminative Thought Style Questionnaire (RTSQ) scores

Model	B	t	P-value	95% confidence interval for B		Adjusted R^2^
				Lower boundary	Upper boundary	
**(Constant)**	49.519	5.514	< 0.001	31.513	67.524	0.156
**Active grief_PGST_3_**	1.325	3.125	0.003	0.475	2.174	
**Family type: large**	15.781	2.031	0.047	0.204	31.357	

Full model includes the following: family type; childlessness; working status; PGST3 (Perinatal Grief Scale T_3_); active grief; difficulty coping; despair.

## DISCUSSION

The aim of this study was to evaluate grief and ruminative thought after a perinatal loss, among Turkish women. This study was specific for perinatal grief in a prospective manner and it makes a valuable contribution to the literature relating to grief, given that this was the first study in Turkey to evaluate grief and ruminative thought style prospectively over a one-year period.

In this study, more than half of the women were experiencing grief at the interview after the first 48 hours. The median values of the grief score periodically decreased over the course of the follow-up measurements.

The grief levels of women who had experienced pregnancy loss have been reported in the literature.^17,21,24–27^ Özgür and Yıldız determined in their study conducted among women over the first three months after pregnancy loss that the average total PGS score did not change within three months.^17^ However, the majority of the studies showed that women experienced various level of grief right after a perinatal loss, and that their grief tended to decrease gradually.^21,24–27^ In a qualitative study, Avelin et al. reported that after the loss of a baby, couples stated that they were still crying and experiencing physical pain at the third month after the loss. On the other hand, one year later, they felt stronger.^26^ This result indicated that couples experience active grief within the first three months and that their feelings change positively over time. The results from many studies have indicated that there are high levels of grief among women who recently had the loss of a pregnancy, and that perinatal grief decreases over time.^28–31^

The results from the current study are in line with data in the literature. In Turkish society, individuals believe that they have something in their destiny and that this cannot be changed. According to faith in one’s destiny, the time of death and the time of marriage are predetermined; therefore, individuals do not have control over them. In Turkish culture, pregnancy or having a baby is blessed. When it comes to losing a most precious thing that people can ever have, society shares all the sad feelings of women and supports them. Accepting their own faith and the support receiving from society may help women to accept their loss and provide positive feelings.

The age variable had a significant effect on the median PGS total score at T_1_ after the loss. Women aged 20-29 years had higher PGS total scores than other age groups. Robert et al. stated that maternal age was a significant predictor of the level of grief and that there was a negative relationship between maternal age and perinatal grief level.^30^ The results from the current study are consistent with those from that study. At early ages in the cycle of life, individuals may not yet have experienced any loss, and a pregnancy loss might be the first major loss in their lives. Women might become more capable of managing negative emotions as they acquire more experiences of life.

In this current study, it was found out that already having children had a significant effect on the median value of the PGS total score at T_1_. Childless women had higher levels of PGS scores at T_1_. Childlessness has been determined to be an important factor with regard to the duration of perinatal grief.^25^ Moreover, Tseng et al. indicated that being a childless woman is a major risk factor for perinatal grief.^27^ The results from our study were similar to those of all these studies. In Turkish society, individuals believe that women who lost their pregnancy may heal through have another living child. Being childless may cause a higher level of grief than that of women who have living children.

We found that the mean RTSQ score of the women was highest at T_0_. The mean scores on the RTSQ showed decreases over the course of the one year of measurements. Thus, the rumination level was highest in the early period of the loss, and it tended to decrease from high to medium rumination gradually. Rumination is considered a cognitive process, in that it has an important role in various psychiatric disorders such as anxiety and mood state disorders.^23^ Studies have reported that among women the risk of experiencing depression increases when their ruminative tendencies increase.^11,14,32,33^ There are no studies in the literature that have evaluated ruminative thought styles among women after perinatal loss. However, several studies have assessed the correlations between rumination levels and anxiety, depression and psychological parameters. Previous studies have shown that rumination causes depressive symptoms because of negative thinking, weak problem-solving skills, insufficient coping behaviors and lack of social support.^33,34^ Rumination has also been found to have positive correlations with depression, anxiety and negative automatic thoughts, and a negative correlation regarding satisfaction with life.^8,11,13–16,33–35^ Additionally, some studies have found that grief may occur in various pathological forms, and that chronic grief may cause depression, anxiety, phobias, obsessions and psychotic reactions.^36^ Therefore, determining the level of rumination after pregnancy loss is quite important in terms of psychopathological conditions such as depression during the early days after the loss.

The results from this study showed that as rumination increases among women in the early period after their loss, grief also increases. This result was supported by the regression analysis in this study. In this current study, the results showed that almost all the women had recovered by the sixth month after the perinatal loss. However, women who are not recovering from grief present more rumination over time than do women who improve. There are no studies evaluating the relationship between perinatal grief and ruminative thought styles in the literature. However, a positive correlation has been reported in the literature between perinatal grief and depression.^24,29,37^

It has been observed that the majority of the women who experienced perinatal loss showed depressive symptoms.^24,29^ Also, similar studies have shown that the risk of incidence of depression after perinatal loss is high.^25,37^ Determining the relationship between perinatal grief and ruminative thought styles might be a starting point for planning qualitative nursing care aimed at protecting and improving women’s mental health after perinatal loss.

### Limitations

The sample size was limited because of the number of dropouts. For this reason, the study results can be generalized only for this population.

## CONCLUSION

In this study, the levels of perinatal grief and ruminative thought styles among women who experienced pregnancy loss were assessed at the first 48 hours and at three months, six months and one year later. These women had high levels of grief and ruminative thought styles in the first 48 hours. However, their levels of grief and ruminative thought styles tended to decrease over the repeated measurements during the follow-up. Based on the results from this study, the following recommendations can be made:

- Nursing assessments regarding grief and ruminative thought style over the first 48 hours after perinatal loss should be integrated into nursing care for these women.
- In clinics, nurses should be trained regarding to how to approach these women, especially during the first 48 hours after the loss.
- Grief follow-up programs for these women can be developed through nursing research, especially during the first six months after the loss.
- Institutions should provide counseling services.
